# Compliment to Prof. Austin Flint

**Published:** 1855-06

**Authors:** 


### EDITORIAL DEPARTMENT.

*Compliment to Prof. Austin Flint.*—The handsome lithograph which accompanies this number, has a history, which will, we believe, add not a little to its value with our old subscribers.

When it became known that our former associate was about to retire from his position as Senior Editor of this Journal, a desire was immediately manifested among his old friends in this city to present to him some testimonial of their appreciation of his long and able discharge of his editorial duties, and of his efficient services in elevating the character of medical literature and science.

This desire has resulted in the lithograph we are now privileged to present to our subscribers. Dr. Flint, during ten years of editorial life has written few lines which he can wish to recall. While he has ever manifested firmness and decision in advocating the right, whether he was to gain or lose by that advocacy, he has always so tempered his language, and given such evidence of honest sincerity, as to disarm enmity in almost every instance. These features of his editorial conduct have secured to him a large body of friends in the profession of his own city.

It was thought that no better or more appropriate evidence of regard could be devised than to send out a good portrait of him, to each of that large number of men who, for so long a period have been familiar with the products of his pen.

A meeting of physicians was accordingly held on the evening of Saturday, April 21, at the office of Prof. James P. White. It was numerously attended. Dr. White was called to the chair, and, after a brief explanation of the objects of the meeting, Drs. T. T. Lockwood, Charles H. Wilcox, and J. B. Samo, were appointed to call on Dr. Flint, and request him to allow Mr. R. J. Compton, of this city, to execute a lithographic portrait of himself "for distribution and preservation among his old friends, and the old subscribers of the Buffalo Medical Journal."

The result is before us. This portrait goes out to the profession at large as a present from the profession in Buffalo, and as an evidence of the feelings with which Dr. Flint is regarded here at home, where he is best known and best esteemed.

By the very liberal action of the subscribers to the portrait fund, we have been permitted to have an extra number of copies struck off for our new subscribers. Mr. Compton has, as usual, presented an excellent likeness. The resemblance is striking, and the artistic execution is of a high order of merit.

